# Transitioning to long-term care for older adults with intellectual disabilities: A concept analysis

**DOI:** 10.1177/17446295211041839

**Published:** 2021-11-10

**Authors:** Caroline Egan, Helen Mulcahy, Corina Naughton

**Affiliations:** University College Cork, Ireland; University College Cork, Ireland; University College Cork, Ireland

**Keywords:** intellectual disability, concept analysis, transition, older adults, long-term care

## Abstract

**Aim:**

To undertake a concept analysis of transitioning to long-term care for older adults with intellectual disabilities.

**Background:**

Individuals with an intellectual disability are experiencing increased longevity which is associated with an increase in transitions in later life to long-term care. Their experience of later life transitions is likely to be different to the general older population.

**Methodology:**

Concept Analysis was undertaken using the Walker and Avant framework.

**Results:**

Eight studies met the inclusion criteria. Defining attributes are an older person with intellectual disability; a planned relocation to a long-term care facility; person-centred; and supported decision-making.

**Conclusion:**

There is a dearth of empirical evidence and theorisation on this concept. Transitions of this nature have been inadequately informed by the perspective of the older person with an intellectual disability, and future research and practice requires greater efforts to include their voice.

## Introduction/background

Within the sphere of intellectual disability there is a plethora of research on ageing however, little of this addresses transitions in later life especially involving a change of residence. The focus of this paper is the concept of transitioning in later life, namely to a long-term care setting from the perspective of older people with intellectual disabilities and their caregivers. These transitions are likely to have a significant emotional and physical impact on older people with intellectual disabilities, yet there is limited consideration of these experiences in policy or clinical practice. It is important to note that transitioning of older adults with intellectual disabilities to long-term care facilities is only one paradigm, there is also the philosophy of ‘ageing in place’. This is defined as the ability of the individual to remain at home for as long as possible as an alternative to moving to an institutional setting (Grimmer et al., 2015). Contemporary approaches within the field of intellectual disabilities also identify ‘ageing in place’ as central to their philosophy and service provision (Bigby, 2008). This concept analysis addresses the alternative paradigm of transitioning to long-term care settings. Within this paper a long-term care setting describes a full-time residential facility which provides a broad range of services including personal, medical or social care which supports a person with cognitive or functional limitations to self-care (Zimmerman and Sloane, 2007). Furthermore, people with intellectual disabilities reside in a number of settings including their family home or residential facilities (NIDD, 2017) which they regard as their home. This concept analysis will include all types of residences from which an older person with an intellectual disability may transition to long-term care.

The concept of transitioning in later life is relatively new due to the increased life expectancy of older adults with intellectual disabilities (Dolan et al., 2019; WHO, 2000). Such longevity is the result of advances in both the social and health care experienced by people with intellectual disabilities over the last century (WHO, 2000). Concurrently, increased life expectancy is often associated with a higher prevalence of chronic illness and disability. Several studies reported higher rates of ill-health, chronic conditions and multi-morbidities (Cooper et al., 2015; McCarron et al., 2013), and higher prevalence of early onset dementia among older adults with Down syndrome (Strydom et al., 2010). This is compounded by poor access to health and social care and health promotion services (Williamson et al., 2017) which can accelerate transitions to long-term residential settings.

Similar to the general population, the biological ageing processes, deterioration in long-term conditions, as well as decline in functional and cognitive capability may necessitate transitioning to higher levels of support and care needs (Bigby et al., 2011). This in turn can increase demands on the primary caregiver potentially triggering a transition process. Another significant issue that accelerates a transition for the person with an intellectual disability is ageing family caregivers who may also experience deteriorating health and functional capability and are no longer in a position to provide the level of care required (Dieckmann et al., 2019). This life-long caring role itself can contribute to the carers health and functional decline and is associated with increasing difficulties with care routines (Grey et al., 2018).

Furthermore, the alignment of national policies with international policy and legislation may inadvertently lead to transitions to long-term care. The prioritisation of social inclusion and de-congregating from institutional settings reflects the principles of the United Nations Convention on the Rights of Persons with Disabilities (UN, 2006). The Convention affirms the rights of people, regardless of age, through explicitly recognising their right to choose their place of residence, facilitated by a range of supports to ensure inclusion in their community. However, a paucity of resources to support ageing individuals with intellectual disabilities has resulted in reduced residential placements within traditional disability services and an over-reliance on family caregivers (Brennan et al., 2017; McConkey et al., 2018). Similarly, the influence of frequently changing government policies and funding for access to alternative accommodation significantly impacted choices among family caregivers and older adults with intellectual disabilities (Trip et al., 2019). This may inadvertently lead to increasing numbers of older adults with intellectual disabilities having no choice but to transition to long-term care facilities in the future.

Research has explored the experiences of older adults with intellectual disabilities who reside in long-term care facilities designed for the general population (Bigby et al., 2008; Thompson et al., 2004). Their findings identified that this population were significantly younger than other residents and indicators suggested they experienced a lower quality of life and social exclusion within these facilities. Within the Irish long-term care sector, there is no accurate data on the number of people with intellectual disabilities living in general long-term care facilities largely due to the absence of a formal diagnosis of intellectual disabilities or a lack of previous contact with disability or statutory services. However, data from Germany suggests that these facilities have become a significant residential placement for this population (Dieckmann et al., 2019).

Any conceptualisation of transitioning requires acknowledgement of the seminal work of Meleis’ grand theory of transitioning which is described as ‘a passage from one fairly stable state to another fairly stable state, and it is a process which is triggered by change’ (Meleis, 2010: 11). Transitions are often precipitated by a significant event, resulting in a period of time of disequilibrium, that requires different actions or strategies such as the development of new skills, seeking supports, development of new relationships and finding meaning within the transition. A number of theoretical frameworks were developed based on Meleis’ theory by Schumacher et al. (Cited, Meleis, 2010: 129) and Davies (2005) which conceptualised the experiences of older adults and their family caregivers among the general older population during transitions into long-term care. However, the experiences and responses of older adults with intellectual disabilities relocating to long-term care are likely to be different to the general population, therefore, the author will undertake an analysis of this concept.

## Rationale for undertaking a concept analysis

Transitioning into a long-term care setting has become a lived experience for many older adults with intellectual disabilities (Bigby et al., 2011; Dieckmann et al., 2019; Webber et al., 2014). Although the phenomenon has been explored, there is inadequate conceptual analysis of the attributes, antecedents and consequences. The impetus for this project is to explore this phenomenon through analysing the concept of transition or relocation to a long-term care facility for older adults with intellectual disabilities. It will operationally define the concept for future reviews and research projects and provides a way to identify and express key ideas about the concept and the essence of transitioning into long-term care for an older person with intellectual disabilities. A concept analysis will delineate the professional’s perspective of this concept through identification of attributes of clinical practice which may facilitate a more positive experience of transitioning for the older person.

## Aim of study

To define, explore and clarify the concept of transitioning into long-term care for an older person with an intellectual disability. This was undertaken using the eight-step approach by Walker and Avant (2014).

## Concept analysis

A conceptual framework using the Walker and Avant (2014) approach was adopted to guide analysis on transitions into long-term care facilities for older adults with intellectual disabilities. This approach was selected as it details a framework which allows for a systematic analysis of the inherent attributes, antecedents and consequences of a concept. Furthermore, it facilitates the development of a working definition and identification of cases of the concept in real-life, so it appeals to a broad range of disciplines and individuals. The framework will map the relationship between ageing with an intellectual disability and transitioning to long-term care. It will explore and construct a conceptual model based on the empirical evidence.

The stages of Walker and Avant (2014: 166) include,

1. Selecting a concept.
2. Determining the aims or purposes of analysis.
3. Identifying all uses of the concept that you can discover.
4. Determining the defining attributes
5. Identifying a model case
6. Identifying borderline, related and contrary cases.
7. Identifying antecedents and consequences.
8. Defining the empirical referents

## Search strategy

Walker and Avant (2014) details that analysis must be rigorous and precise. Therefore, all possible uses of the concept of transitions were explored. A systematic search was undertaken, the research question was based on the PICo mnemonic (Stern et al., 2014). PICo with a lowercase ‘o’ can be useful when seeking to analyse human experiences or phenomena. The population of interest were older adults with intellectual disabilities. The phenomenon of interest is transition or relocating to a long-term care facility, including that which predicted it, the consequences of transitioning, and contextual issues such as geographical or residential setting. Keywords relating to transitioning, long-term care and intellectual disabilities were combined (Online Supplemental Tables 1 and 2). The following electronic databases: CINAHL, Medline, Psych Info, (Embase) and Web of Science were searched. A Grey literature search was undertaken using Google Scholar to identify government and health department reports, intellectual disability charity or stakeholder documents and evidence of relevant theses or dissertations. Finally, bidirectional citation searching was undertaken concurrently (Hinde and Spackman, 2015).

Inclusion criteria were studies which: (1) explored actions or experiences of transitioning into long-term care for an older person with an intellectual disability and their caregivers (including family, paid carers); (2) contained definitions or discussed the concept of transitions within this context; (3) identified antecedents or consequences of transitioning to a long-term care facility; (4) detailed the physical, emotional or social effects of transitioning; (5) primary research studies and (6) literature published between the years 2000–2020.

The exclusion criteria were research which (1) explored transitioning to long-term care facilities for older adults within the general population; (2) related to life transitions for children and younger adults with intellectual disabilities; (3) explored ‘ageing in place’ as a strategy to support older adults or deinstitutionalisation to community settings; (4) examined placement of young people with intellectual disabilities in nursing home settings.

**Figure 1.**
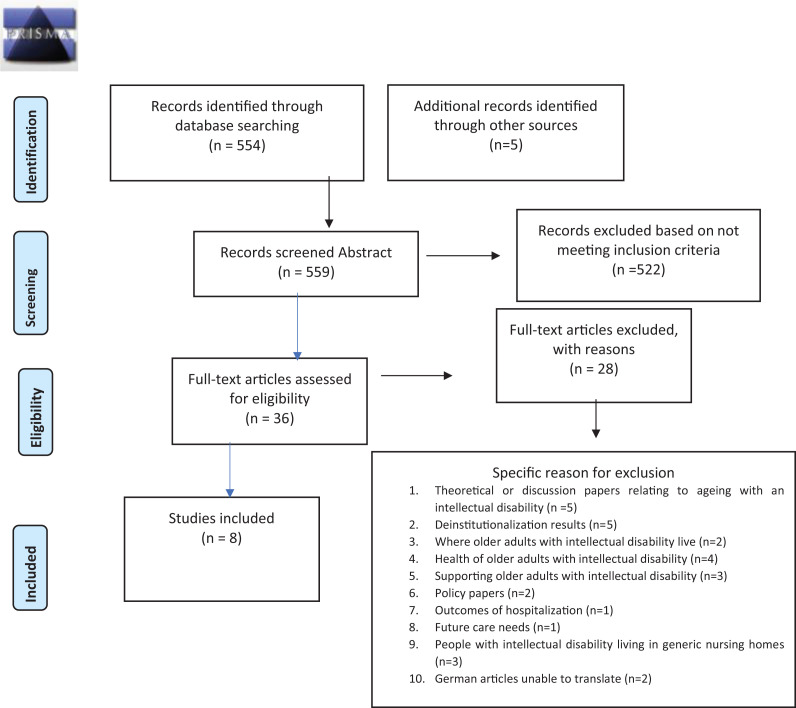
PRISMA 2009 flow diagram.

The searches were undertaken from September to November 2020. The first stage of the search identified 554 papers. Searches of references and grey literature identified five further papers which were included. Following review of abstracts and the removal of duplicates 36 studies were imported into Mendeley referencing system for full text review, from which 8 studies met the inclusion criteria. Two authors CE & HM independently screened the 36 studies and reached consensus on the final 8 included studies. The selected articles were in English and one German study was translated into English. The search is outlined in PRISMA flow chart (2009) (Figure 1).

## Uses of the concept

Of the final eight studies, five were from Australia (Bigby et al., 2011; Buys et al., 2012; Webber et al., 2010, 2014, 2016), two from America (Janicki et al., 2002; Patti et al., 2010), and one from Germany (Dieckmann et al., 2019). The study designs included four qualitative, three quantitative and one mixed-methods. The qualitative papers explored the perceptions of family and paid caregivers’ decisions around transitioning to long-term care facilities for older adults with intellectual disabilities. The main themes centred on decision-making processes, factors leading to and reasons why a transition was imminent (Bigby et al., 2011; Buys et al., 2012; Dieckmann et al., 2019; Webber et al., 2010). Two studies (Dieckmann et al., 2019; Webber et al., 2014) analysed the experiences of older adults who had transitioned from the perspective of their caregivers, while Dieckmann et al. (2019) was the only study to include older adults with intellectual disabilities through making reasonable adjustments including the use of simple language, picture cards and graphics to aid inclusion in interviews. Two studies adopted a quantitative design which measured paid caregivers’ experiences of providing supports to ageing individuals and their attitudes in relation to ageing in place or progression to long-term care facilities (Janicki et al., 2002; Webber et al., 2016). The final study used a retrospective cohort approach to investigate associations between ageing with an intellectual disability and relocation to a long-term care facility (Patti et al., 2010).

## Defining attributes

The papers provided little theorisation on the concept itself however, with transitioning identified as a central concept. Bigby et al. (2011: 779) defined the concept as the older adult with an intellectual disability who ‘experiences age associated changes to health and functional capacity’…. The transitional point ‘at which decisions are made that a person can no longer be supported’ to age in place within their home and moves to a long-term care setting.

Four defining attributes or characteristics repeatedly appear in the literature (Table 1)

- An older person with an intellectual disability.
- A planned relocation to a long-term care facility more appropriate to their ageing needs.
- Person-centred.
- Supported decision-making.

**Table 1.** Literature supporting the defining attributes.

Attributes	References
An older person with an intellectual disability	Bigby et al. (2011); Buys et al. (2012); Dieckmann et al. (2019); Janicki et al. (2002); Patti et al. (2010); Webber et al. (2010, 2014, 2016)
A planned relocation to an long-term care facility more appropriate to their ageing needs	Bigby et al. (2011); Buys et al. (2012); Dieckmann et al. (2019); Janicki et al. (2002); Patti et al. (2010); Webber et al. (2010, 2014, 2016)
Person-centred	Bigby et al. (2011); Buys et al. (2012); Dieckmann et al. (2019); Janicki et al. (2002); Webber et al. (2010, 2014, 2016)
Supported decision-making	Bigby et al. (2011); Dieckmann et al. (2019); Janicki et al. (2002); Webber et al. (2010, 2014, 2016)

The first defining attribute is the ***older adult with an intellectual disability*.** An intellectual disability is defined as a disability characterised by significant limitations in both intellectual functioning and adaptive behaviour, which covers many everyday social and practical skills originating before the age of 22 years (American Association of Intellectual and Developmental Disabilities, 2010) or 18 years (American Psychiatric Association, 2013). Clarity on what defines an older adult with an intellectual disability is less obvious. Older adults with a mild intellectual disability in western countries have a similar life expectancy as the general population (WHO, 2000). However, people with specific syndromes, such as Down syndrome, profound intellectual disabilities or complex and multiple disabilities have a reduced life expectancy (WHO, 2000). Premature ageing is evident in some cohorts including cognitive and functional decline with high rates of geriatric conditions e.g. falls, immobility, incontinence evident from as early as 40 years (Carfi et al., 2019; WHO, 2000). Additionally, premature ageing is associated with high rates of frailty among those with intellectual disabilities (McKensie et al., 2017). Consequently, ageing can occur well in advance of chronological age. Therefore, older adults with intellectual disabilities will be defined as individuals over 40 years of age who present with health and functional decline associated with ageing processes.

The second attribute is ***A planned relocation to a long-term care facility which is more appropriate to their ageing needs***. A planned relocation is a permanent move into a long-term care facility made with foresight and in a well-informed manner (Dieckmann et al., 2019). A long-term care facility is described as a skilled nursing facility which provides a range of personal and health care with a focus on medical, nursing care, safety and support with activities of daily living (National Institute on Ageing, 2020). Needs are associated with ageing processes such as developing a chronic health condition or experiencing functional decline (Bigby et al., 2011; Webber et al., 2014, 2016).

***Person-centred*** is the third attribute. The American Geriatric Society (2015) expert panel describes person-centred care as the recognition and engagement with the person’s values and preferences, which are used to guide care. Person-centred care is demonstrated through the establishment of a therapeutic relationship, sharing of power and responsibility, empowerment, trust, respect and communication (Sharma et al., 2015). Furthermore, in the non-acute setting person-centred care was conceptualised by Morgan and Yoder (2012) as synonymous with a respect for several ethical values including approaches that are holistic, individualised, respectful and empowering. The analysis, presented approaches which were person-centred. Therapeutic long-term relationships between the older person and their family or professional caregivers were a cornerstone of studies. Person-centred approaches were empowering, respectful and trusting. Furthermore, caregivers recognised that holistic, individualised care which considered the whole person was essential. Person-centred approaches encompassed advocacy, beneficence, autonomy and responsibility. Family caregivers voiced feelings of responsibility to advocate on behalf of their family member with an intellectual disability (Bigby et al., 2011; Webber et al., 2014). Equally, professionals aspired to do their best to advocate for the person with an intellectual disability. Planning and actions came from altruistic concern and wanting to do one’s best for the person reflective of a person-centred philosophy.

The fourth attribute is ***supported decision-making*** which involves individual’s working together supporting the older person with an intellectual disability to make informed choices from all available options. Transitioning decisions within the studies were made by family members or disability staff (Bigby et al., 2011; Dieckmann et al., 2019; Janicki et al., 2002; Webber et al., 2010, 2014, 2016). Individuals with intellectual disabilities were included as participants in Dieckmann et al. (2019) study however, they did not experience themselves as decision-makers but rather submitted to the advice of others including disability staff and family caregivers. The UNCRPD (UN 2006) Article 12 requires signatory countries to move towards supported decision making approaches. Future approaches to transitional decisions must enable the individual to exercise their legal capacity to the greatest extent in transitional decisions according to their wishes. Furthermore, the convention further asserts that the processes are put in place to ensure that the person’s voice is evident (UN, 2006). Such approaches may include shared decision-making or a representative agreement within a support network. Supported decision-making was advocated for to support self-determination of the older adult with an intellectual disability and family involvement in transitions (Bigby et al., 2011; Dieckmann et al., 2019; Webber et al., 2014).

## Definition

A definition is proposed based on the defining attributes and analysis of this concept namely,

**A supported decision involving an older person with an intellectual disability is made to facilitate a planned relocation to a long-term care facility which reflects a person-centred philosophy.**

## Antecedents

The antecedents included health and functional decline as a result of ageing, staff knowledge and competencies, family caregiver issues, disability service inflexibility/inappropriate fit and organisational issues (Table 2). Similar to the general population, older adults with intellectual disabilities may develop health conditions or experience a decline in functional capacity precipitating a relocation to long-term care (Bigby et al., 2011; Buys et al., 2012; Dieckmann et al., 2019; Janicki et al., 2002; Webber et al., 2010, 2014, 2016). Furthermore, people with congenital syndromes, such as Down syndrome, experienced accelerated ageing and were predisposed to relocating to a long-term care facility due to functional decline (Patti et al., 2010). Several studies explored the impact of ageing processes on residential services which prompted a move to long-term care. Bigby et al. (2011) and Webber et al. (2016) reported that staff in group homes were ill-prepared to support the person with newly emerging health needs or functional decline. A lack of training and poor staff understanding was further compounded by organisational issues including insufficient fit and adaptation to the newly emerging needs of the older adult which made ‘ageing in place’ difficult (Dieckmann et al., 2019; Webber et al., 2016). Further examples of inadequate staff support and skills included absence of 24-hour care, lack of qualified staff to support complex health interventions and inflexible day care services. This resulted in an overburdening of disability services which further impacted the lives of other group home residents (Bigby et al., 2011; Webber et al., 2010). The philosophy of more experienced or managerial disability staff had a significant impact on whether the person should stay or move to a long-term care setting (Webber et al., 2010). Additionally, inflexible organisation funding rules precipitated a move (Janicki et al., 2002). Indeed Webber et al. (2010) highlighted a lack of funding and staffing models to appropriately meet the ageing needs of this population within disability settings such as one-to-one care provision. Dieckmann et al. (2019) reported that family caregiver wellbeing was identified as another antecedent to relocating from the family home to long-term care namely, when the primary caregiver was no longer able to continue caring due to frailty or death of the primary caregiver.

**Table 2.** Literature support for the antecedents.

No.	Antecedents	Literature
1	Health and functional decline	Bigby et al. (2011), Buys et al. (2012), Dieckmann et al. (2019), Janicki et al. (2002), Patti et al. (2010), Webber et al. (2010, 2014, 2016)
2	Disability service inflexibility/inappropriate fit	Bigby et al. (2011), Dieckmann et al. (2019), Janicki et al. (2002), Webber et al. (2010)
4	Staff knowledge and skills	Bigby et al. (2011), Dieckmann et al. (2019), Janicki et al. (2002), Webber et al. (2010, 2014, 2016)
5	Family challenges	Dieckmann et al. (2019)

## Consequences

The studies presented both positive and negative consequences of transitioning to long-term care facilities (Table 3). Positive experiences were associated with overall improvements in the person’s health and wellbeing following relocation (Webber et al., 2014). Family and disability services identified that positive experiences were influenced by satisfaction with how decisions were made (Bigby et al., 2011). Responsiveness to the needs of the individual and their caregivers was positively received, indeed, families noted that placement in a facility near the family home was very important to maintain contact (Dieckmann et al., 2019). Planning which maintains social relationships and a sense of connection with former relationships was viewed positively; interestingly ‘group relocations’ was presented by both Bigby et al. (2011) and Dieckmann et al. (2019) as an option. A group move involves existing residential groups who are simultaneously ageing and experiencing both health and functional decline, relocating together to a long-term care facility. In the studies reviewed, positive experiences were associated with the perception that the older person with an intellectual disability was happy, settled, connected to their former life and in the correct place to meet their needs.

**Table 3.** Literature support for the consequences.

No.	Consequences	Literature
1	**Positive experiences** Health and wellbeing improvements Satisfaction with decisions making Responsiveness	Bigby et al. (2011); Deickmann et al. (2019); Webber et al. (2014)
2	**Negative experiences** Social isolation Lack of involvement in decisions Disconnection with past Lack of knowledge	Bigby et al. (2011); Dieckmann et al. (2019); Webber et al. (2014)

Negative experiences were reported by Bigby et al. (2011), Dieckmann et al. (2019) and Webber et al. (2014). Families reported a lack of involvement in the whole decision-making process, that decisions were hastily made, and they felt unprepared due to a lack of knowledge of the inherent rights of the person with an intellectual disability (Bigby et al., 2011). Dieckmann et al. (2019) reiterated this lack of involvement in decisions with participants noting an absence of neutral advice and alternative housing choices for ageing people with intellectual disabilities. Family members and support staff identified that social isolation was experienced by older adults with intellectual disabilities post-transition (Webber et al., 2014). This was compounded by a lack of acceptance of people with intellectual disabilities among the other residents in long-term care. Staff observed that residents were often afraid or uncomfortable with residents with intellectual disabilities and avoided them (Webber et al., 2014). Similarly, disability staff consistently voiced concern that long-term care staff did not understand the social needs of this population citing inappropriate staffing and activities programmes as well as disciplinary differences between staff in disability and long-term care settings (Buys et al., 2012). Families reiterated concerns about the disconnection from past relationships, hobbies and the lack of interaction post transition to long-term care (Webber et al., 2014).

**Figure 2.**
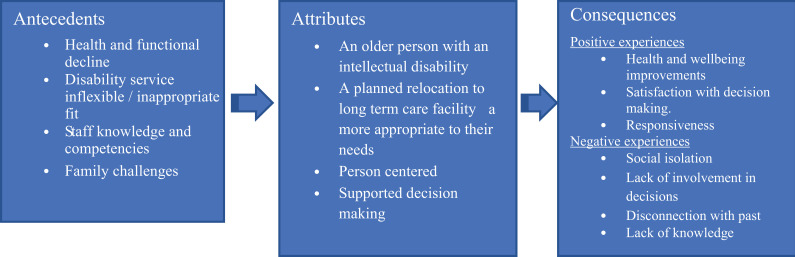
Transitioning to aged care for older adults with intellectual disabilities: A conceptual framework.

The defining attributes, antecedents and consequences are presented in a conceptual framework (Figure 2)

## Case examples

### Model case

The model case is a real-life example which includes all the defining characteristics. They are hypothetical but have been informed by the author’s nursing experiences supporting older adults with intellectual disability.

John is a 60-year-old man with Down syndrome and a diagnosis of Alzheimer’s dementia. He previously resided in a disability group home which was staffed during the day with one person sleeping over at night. John frequently wandered at night and spent his days rummaging through his personal artefacts in his bedroom. When John was asked to attend his day service or go to bed at night, he became distressed and refused, causing stress to the other residents, sleepover staff and John. The supports offered by the disability service were failing to meet John’s functional decline and changing needs. John appeared unhappy and it was decided with John that supporting his relocation to an appropriate long-term care facility nearby may suit John better. A transition plan was created in collaboration with John, his sister, long-term care and disability staff. A planned relocation over 3 months involved visiting the long-term care facility on a weekly basis enabling long-term care staff to meet and get to know John, his sister and group home staff. This ensured that long-term care staff become familiar with his individualised needs and supports. A social timetable was established so former residents could visit and maintain social contact with John thus maintaining a link with his past-life. John transitioned over a 3-month period and settled well into his new home. A health assessment identified several health deficits with interventions implemented. Presently, John continues to maintain friendships from his previous life and enjoys activities within the long-term care facility. The transition plan was evaluated by John who reported that he feels *happy and is enjoying his new home.*

The model case includes all the defining attributes. John, an older gentleman with an intellectual disability, was experiencing both health and functional decline due to a diagnosis of dementia. A planned relocation was created involving his relocation to an long-term care facility nearby. John, his sister and disability support staff created a person-centred transition plan. Supported decision-making through meaningful efforts were made to include John’s wishes and to maintain the connection with his previous life. The transition was responsive to John’s changing needs and demonstrated collaboration between parties.

### Additional cases

#### Borderline case

Borderline cases contain most of the defining attributes but are inconsistent with the present concept.

Mary is a 63-year-old lady with a moderate intellectual disability who previously lived in a group home. Mary was diagnosed with Parkinson’s disease and had fallen several times. The group home vacated during the day with residents attending the day services. Mary was exhausted and verbalised that she would like to reduce the time she spent at the day service. There were no staff available to support Mary if she remained at home and Mary required ongoing support throughout the day. Her elderly parents and support services were anxious that Mary relocated to a nearby nursing home where she could be supported. Staff and her family recognised that talk of a relocation was upsetting Mary and created a transition plan without her involvement. Long-term care, disability staff and Mary’s family met and had a case conference. A planned relocation involved Mary moving to long-term care was initiated, her keyworker was allocated 10 hours a week for 2 months to work as a personal assistant to ensure Marys’ social and psychological needs were supported during the relocation. This ensured that staff became familiar with her individual needs and supports. Provisions were made for Mary to continue to attend her day service for half day sessions if she so wishes following her move to long-term care.

This case example represents a borderline case. Mary, an older adult with an intellectual disability, was experiencing both health and functional decline. A transition plan was created to facilitate a relocation to a long-term care facility nearby. The attribute of supported decision-making is inconsistent with the concept as Mary was not involved in the planning nor did it provide Mary with opportunities to self-determine. A person-centred transition plan was created with efforts to advocate for Mary and to maintain the connection with her previous life.

#### Related case

Related cases are instances of the concept which are related but do not contain all the attributes.

Deirdre is a 65-year-old lady with a mild level of intellectual disability. She previously lived with her brother who suffered a stroke, he could no longer meet her needs and decided to relinquish care. There were no available residential beds within the local disability service which Deirdre attended therefore out of necessity Deirdre relocated directly into a long-term care facility near her brother’s home. Deirdre was nervous but settled very quickly into her new routine. Deirdre received a full health checkup and was commenced on a modified diet. She lost weight and reported she feels overall much better. Over time, Deirdre lost contact with her previous friends and interests.

This case does contain some of the attributes, but they differ when examined closely. Deirdre is an older adult with an intellectual disability however, she was not experiencing health and functional decline associated with ageing processes. The relocation to a long-term care facility was not planned. The attribute of supported decision-making is inconsistent with the concept as Deirdre was not involved in the planning, the move was reactive and there was no time to support her involvement in decision making. A lack of choice and resources restrict available options therefore, a person-centred plan did not feature as central in this relocation.

#### Contrary case

Is not an example of the concept

Eddie is a 74-year-old man without an intellectual disability, but with a diagnosis of dementia. His dementia had progressed quite rapidly and following a period of hospitalisation for an acute illness, it was decided that he would not return home and was relocated to long-term care facility. Eddie was saddened and became depressed, isolated himself refusing to leave his bed following the relocation.

This is an example of a contrary case and is clearly not an example of the concept. Eddie is not an older adult with an intellectual disability, nor is the relocation planned. Eddie was discharged to the first available long-term care facility as there was no family at home to care for him. The relocation did not prioritise Eddie’s needs nor was it person-centred as the focus was on a timely discharge from acute care. There were no opportunities for Eddie to self-determine or his involvement in relocation decisions. Once Eddie was relocated there was an absence of attention to his social and psychological needs.

## Defining the empirical referents

The empirical referents are a way to measure and recognise the concept of transitioning into long-term care for an older adult with an intellectual disability in both practice and research. Exemplars of alder adults with intellectual disabilities and planned relocation to long-term care facilities more appropriate to their needs, were represented in the demographic details of the referrent studies (Bigby et al., 2011; Buys et al., 2012; Dieckmann et al., 2019; Janicki et al., 2002; Patti et al., 2010; Webber et al., 2010, 2014, 2016). Decision-making within the context of transitioning was measured using several methodologies. Three of the studies adopted qualitative approaches using grounded dimensional analysis (Bigby et al., 2011; Webber et al., 2010, 2014). This methodology involved approaches which explored complex phenomena, early interviews were exploratory, evolving over time to become focused. Theoretical sampling was undertaken to direct later interviews and subsequently develop theory on the decision-making processes and actions of transitioning older adults with intellectual disabilities. Quantitative measurement of decision-making was described by Janicki et al. (2002), this involved a postal survey of 54 group homes serving at least one older adult with an intellectual disability who were experiencing health and functional decline due to dementia. The survey examined data related to decision-making namely, whether the older person would ‘age in place’ or progress to a specialised long-term care facility. One study (Dieckmann et al., 2019) adopted a mixed-methods approach, quantitative telephone interviews and surveys were completed in 22 long-term care facilities which were home to older adults with intellectual disabilities. This was further explored using qualitative interviews within three long-term care facilities. Data were collected on demographic information of older residents with intellectual disabilities, their reasons for relocating and their involvement in decision-making.

A person-centred approach was an observable phenomon evident in the included studies (Bigby et al., 2011; Buys et al., 2012; Dieckmann et al., 2019; Webber et al., 2010, 2014). This attribute was not directly measured in the included studies but was thematically extracted during this concept analysis.

Health and functional decline as an antecedent to a planned relocation to a long-term care facility was explored by Webber et al. (2016) who undertook an online survey of 76 group home care staff, examining their training and confidence in meeting the health needs of older adults and attitudes towards relocating adults with intellectual disabilities to long-term care. Other qualitative approaches involved semi-structured interviews (Buys et al., 2012), which reported on the attribute of a planned relocation to a long-term care setting due to health and functional decline of an older adult with an intellectual disability.

## Discussion

The present analysis addresses the concept of transitioning for older adults with intellectual disabilities to a long-term care setting from the perspective of caregivers rather than the older person themselves. From the included papers the defining attributes are, an older person with intellectual disability, a planned relocation to a long-term care facility more appropriate to their ageing needs, person-centred approaches to identify wishes and preferences and supported decision-making. To date the concept has received little attention leading to a dearth of empirical evidence and theorisation. A significant gap in the literature when defining the concept is the absence of the voice of older people with intellectual disability.

Transitions among older adults with intellectual disabilities was explored by Strnadová (2019) who presents types of transitions in later life involving relationships, residential, work, retirement, and health. As far as the author is aware, this is the only concept analysis to address residential transitions in later life experienced by individuals with intellectual disabilities.

Supported decision-making was identified as a defining attribute which involves individual’s working together supporting the older person with an intellectual disability to make informed choices from all available options. However, the evidence suggested a lack of self-determination on the part of the older adult with an intellectual disability (Bigby et al., 2011; Dieckamnn et al., 2019; Webber et al., 2014). Critically, decisions did not involve the older person in a meaningful or acceptable way. Therefore, the concept is not adequately informed by the views of the people most centrally involved in the experience. Future research should include people with intellectual disabilities not only as participants, but also as collaborators and co-researchers. Researchers should link with advocacy groups to ensure accessible information in the form of interviews and ethical considerations to ensure meaningful inclusion using sensitive approaches (Herron et al., 2015), as the views of those most impacted by these transitions are paramount. There is a need to develop creative and inclusive methodologies to include older adults with intellectual disabilities in research which addresses their rights and creates a platform for their voices to be heard in a non-tokenistic way (Salmon et al., 2018).

Several of the studies noted the different care philosophies and competencies between long-term care and disability staff (Webber et al., 2010, 2014). Greater collaboration between long-term care and disability services may facilitate decisions-making processes and shared learning. Such integrated approaches could potentially champion supportive decision-making which may improve the experiences of all those involved and assist in the implementation of holistic person-centred transitioning plans. Heller (2019) and Trip et al. (2019) advocated for greater collaboration between long-term care and disability services whereby the expertise of both can enhance the quality of care including bridging ageing and intellectual disability in research, policy and practice.

A person-centred approach to care was identified as a defining attribute. This was characterised by approaches which empower, respect and involve the person in relocation decisions. However, the included studies presented findings which presumed that the older person lacked capacity and thus precluded the person in relocation planning or choices. This raises concerns regarding how we involve people with intellectual disabilities in such transitional processes. Transition planning or actions should reflect the human rights of this population (UN, 2006) therefore, researchers and disability services have an important role to ensure that the voice of people with intellectual disabilities is evident throughout the processes and findings. Older adults with intellectual disabilities would also benefit from regular and accessible education to ensure health literacy, life skills and that people with intellectual disabilities are aware of their rights, which may facilitate choice and access (Trip et al., 2019). Furthermore, internationally, countries are recognising and passing laws to legalise frameworks which recognise the person’s right regardless of disability to make such decisions (UN, 2006). In Ireland, legislation was updated through the Assisted Decision-Making (Capacity) Act ( Government of Ireland, 2015). This law integrates the human rights approaches of self-determination, choice and consent placing an onus on society and health and social services to facilitate a person’s ability to make their own decisions about their lives including decisions in respect to transitioning in later life. Such transitions can benefit from inclusion of independent advocates and integrating individual-level reasonable adjustments (Heslop et al., 2019) to ensure the older adult with an intellectual disability is included and can participate to the fullest extent possible in decisions.

Facilitating ‘ageing in place’ for individuals with intellectual disabilities is central to many services and policy (Bigby, 2008). However, the studies included in this analysis, demonstrated that service providers and staff experience difficulties in responding to the needs of the older person with an intellectual disability within existing residential settings (Bigby et al., 2011; Webber et al., 2010, 2016). In the short term, It is evident that there will continue to be a need for transitions and thus the need to identify best practice on how to support those who are or will relocate to a long-term care setting in the future. Furthermore, a clear lack of guidance, planning and education was evident from direct care staff to organisational decision makers (Bigby et al., 2011; Webber et al., 2010). Subsequently disability staff were ill-equipped to meet the newly emerging needs of this population which influenced decisions to move residents to a long-term care facility. Decisions were made with staff citing a duty of care or recognition that the current setting was ill equipped to meet their progressive care needs. However, there was uncertainty as to what indicators or triggers signalled when a transition was imminent. There is a need for evidence-based policy and guidelines at organisation, governmental and professional level to inform these decisions. This is in-keeping with professional caregiver’s experiences of caring for older adults with intellectual disabilities, the need for effective planning and integrated care was highlighted by Doody et al. (2012). Such approaches may enhance responsiveness and person-centred care.

Transition to long-term care was not always associated with negative experiences, none the less, the majority of studies tended to dwell on the negative consequences (Dieckmann et al., 2019; Webber et al., 2014). Social isolation as a negative consequence of relocating to a long-term care setting was identified by family members and support staff (Webber et al., 2014). This was compounded by a lack of acceptance of people with intellectual disabilities by the other residents in long-term care. These findings are in-keeping with data from a large national representative sample of people with disabilities, Emerson et al. (2020) reported higher levels of loneliness and social isolation among people with intellectual disabilities compared to their non-disabled peers. Non-specialist long-term care facilities make well intentioned efforts to include the older person with an intellectual disability as part of the care home community but a lack of adequate understanding of the their unique needs may result in limited success (Webber et al., 2014). There is an onus on professionals and services to develop social inclusion strategies as part of a transition plan for older people with intellectual disabilities. Approaches should explore strategies to maintain past relationships, hobbies and interests. Indeed, Webber et al. (2014) advocated for the development of expertise in working with people with intellectual disabilities in long-term care facilities. Concurrently, educational institutions and disability services could develop expertise and services to facilitate person-centred transitional care for older adults with intellectual disabilities.

## Implications for practice

A deeper understanding of the transitional process associated with relocating to a long-term care settings may improve the experiences of caregivers and the older adult with an intellectual disability, during this stressful life event. Future research and practice development need to prioritise the development of strategies to promote self-determination and supported decision-making relating to relocation decisions. The analysis has identified elements of a person-centred transition that could be used as a guide to practice. Furthermore, long-term care facilities may learn from contemporary approaches to reduce social isolation including buddy services or the development of structured social groups involving the person with an intellectual disability (Wilson et al., 2016) and flexibility in attending or visiting day services or previous residences. In the short term, we need to develop evidence based organisational and professional policies, guidelines and staff competencies to support a person-centred relocation model. In the longer term, intellectual disability services need to examine pathways to ‘age in place’ to facilitate choice and preferences of older people with intellectual disabilities and families. Consideration of these practices need to be addressed on a case-by-case basis reflective of a person-centred philosophy.

## Conclusion

This paper operationally defines the concept for future reviews and research projects through providing a definition, identifying key ideas and findings from the evidence base while delineating the concept of transitioning into long-term care for an older person with an intellectual disability. Future research involving older adults with intellectual disabilities, and integration of this into the concept, will facilitate a more rounded concept.

Older people with intellectual disabilities are and will increasingly transition to some category of long-term care setting in the future. This concept of transitioning was analysed from the limited literature on the topic. Critically the concept analysis findings do not reflect contemporary approaches including person-centredness or the ethical standards which we accept as human rights for all people. We as a society including individuals with intellectual disabilities, their family caregivers, disability staff and services, researchers and academics must do better to reflect the voice of those most impacted by these transitions.

Future research involving older adults with intellectual disabilities, their wishes and experiences relating to transitions is required to inform this concept. Evidence based practice and policies based on principles of inclusion, self-determination, shared and supported decision-making in line with mental capacity legislation is essential. Intellectual disability services also require standardised and evidence based pathways appropriately resourced to enable person-centred transitions to improve experiences and quality of life of older people with intellectual disabilities.

## Supplemental Material

Supplemental Material, sj-docx-1-jld-10.1177_17446295211041839 - Transitioning to long-term care for older adults with intellectual disabilities: A concept analysis Click here for additional data file. Supplemental Material, sj-docx-1-jld-10.1177_17446295211041839 for Transitioning to long-term care for older adults with intellectual disabilities: A concept analysis by Caroline Egan, Helen Mulcahy and Corina Naughton in Journal of Intellectual Disabilities
