# Supplementary material for: Transitioning to long-term care for older adults with intellectual disabilities: A concept analysis
Source: J Intellect Disabil. 2021 Nov 10;26(4):1015–32. doi: 10.1177/17446295211041839 (PMC9607934; doi:10.1177/17446295211041839)
Supplement: Supplemental Material, sj-docx-1-jld-10.1177_17446295211041839 - Transitioning to long-term care for older adults with intellectual disabilities: A concept analysis [file sj-docx-1-jld-10.1177_17446295211041839.docx]

Table [1](#HueD_Tab2). Database searches: CINAHL, PsychInfo and Medline (3/11/2020).

| S1  AB, TI SU  Transition* or relocat* or chang* or transfer* or mov* or transfigur* or progress* or shift* or switch* or graduat* or relinquish*  Hits = (6,852,667)  S2  ‘Aged Care Facilit*’ OR ‘Aged Residential Care’ OR ‘Assisted Living Facilit*’ OR ‘Care Home*’ OR ‘Community Hospital*’ OR ‘Continuing Care’ OR ‘Elder Care’ OR ‘Geriatric Care Facilit*’ OR ‘Gerontolog* Care’ OR ‘Long term care’ OR ‘Long Stay’ OR ‘Nursing Home*’ OR ‘Residential Aged Care Facilit*’ OR ‘Residential Care Home*’ OR ‘Skilled Nursing Facilit*’ OR ‘Supported Care Facilit*’ Or ‘psychogeriatric unit’ or ‘developmental centre’  Hits = (229,971)  S3  AB, TI SU  ‘Intellectual Disability’ or ‘Developmental disability’ or ‘Learning Disability’ or ‘Mental Handicap’ or ‘Down Syndrome’ or ‘trisomy 21’ or ‘Cerebral Palsy’ or Autis* or Intellectual N3 impairment* or Intellectual N3 disabilit* or Intellectual N3 delay* or Intellectual N3 handicap* or Intellectual N3 retard* or mental N3 Impair* or mental N3 disabilit* or mental N3 disable* or mental N3 handicap* or mental* N3 delay* or developmental* N3 Impair* or developmental* N3 disabil*or  developmental* N3 disable* or developmental* N3 handicap* or developmental* N3 delay* or learning N3 impair* or learning N3 disabil* or learning N3 disable* or learning N3 handicap* or learning N3 delay*  Hits – (402,089)  Combine with ‘and’ Hits = 441  2000–2020 = 314. |
| --- |

Table 2. Database searches: Web of Science (6/11/2020).

| Search 1  Transition* or relocat* or chang* or transfer* or mov* or transfigur* or progress* or shift* or switch* or graduat* or relinquish*   TOPIC = (11,847,091)  &  Search 2  ‘Intellectual Disability’ or ‘Developmental disability’ or ‘Learning Disability’ or ‘Mental Handicap’ or ‘Down Syndrome’ or ‘trisomy 21’ or ‘Cerebral Palsy’ or Autis* or ‘Intellectual NEAR/3 impairment*’ or ‘Intellectual NEAR/3 disabilit*’ or ‘Intellectual NEAR/3 delay*’ or ‘Intellectual NEAR/3 handicap*’ or ‘Intellectual NEAR/3  retard*’ or ‘mental NEAR/3  Impair*’ or ‘mental NEAR/3 disabilit*’ or ‘mental NEAR/3  disable*’ or ‘mental NEAR/3 handicap*’ or ‘mental* NEAR/3 delay*’ or ‘developmental* NEAR/3  Impair*’ or ‘developmental* NEAR/3 disabil*’ or ‘developmental* NEAR/3  disable*’ or ‘developmental* NEAR/3 handicap*’ or ‘developmental* NEAR/3  delay*’ or ‘learning NEAR/3 impair*’ or ‘learning NEAR/3 disabil*’ or ‘learning NEAR/3 disable*’ or ‘learning NEAR/3 handicap*’ or ‘learning NEAR/3 delay*’  TOPIC = (157,495)  &  Search 3  ‘Aged Care Facilit*’ OR ‘Aged Residential Care’ OR ‘Assisted Living Facilit*’ OR ‘Care Home*’ OR ‘Community Hospital*’ OR ‘Continuing Care’ OR ‘Elder Care’ OR ‘Geriatric Care Facilit*’ OR ‘Gerontolog* Care’ OR ‘Long term care’ OR ‘Long Stay’ OR ‘Nursing Home*’ OR ‘Residential Aged Care Facilit*’ OR ‘Residential Care Home*’ OR ‘Skilled Nursing Facilit*’ OR ‘Supported Care Facilit*’ Or ‘psychogeriatric unit’ or ‘developmental centre’  TOPIC = (75,684)  Combine with ‘and’ Hits = 113 |
| --- |
